# A Case of Brain Tuberculosis With an Unusual Presentation Mimicking Viral Fever During a Dengue Outbreak in Bangladesh

**DOI:** 10.7759/cureus.21260

**Published:** 2022-01-15

**Authors:** Muhammad Rezeul Huq, M. A. Hannan, Shahida Bulbul, Anis Ahmed, Ahad Mahmud Khan

**Affiliations:** 1 Department of Neurology, Combined Military Hospital, Dhaka, BGD; 2 Department of Neurology, Bangabandhu Sheikh Mujib Medical University, Dhaka, BGD; 3 Epidemiology, Projahnmo Research Foundation, Dhaka, BGD; 4 Global Health, Usher Institute, The University of Edinburgh, Edinburgh, GBR

**Keywords:** dengue mimics, tuberculosis mimics, central nervous system, brain, tuberculosis

## Abstract

Both dengue and tuberculosis are endemic in South Asian countries, including Bangladesh. Here we report an interesting case presenting as suspected dengue fever and eventually diagnosed as a case of brain tuberculosis. A 27-year-old immunocompetent male presented to us with fever, headache, retro-orbital pain, and photophobia for five days. He had no other complaints, and clinical examination findings were normal. Full blood count revealed neutrophilic leukocytosis; dengue antigen test and anti-dengue antibody test were negative. Magnetic resonance imaging (MRI) of the brain showed both supra and infra-tentorial multiple small (2-4 mm) gadolinium-enhancing lesions suggestive of tuberculomas. A cerebrospinal fluid study revealed lymphocytic pleocytosis with raised protein, low sugar level, and positive Gene Xpert MTB/RIF (Cepheid, California, US) assay test. Investigations did not reveal the involvement of other organs except for the brain. We started standard anti-tuberculosis therapy (HRZE) along with steroids and pyridoxine, and the patient became symptom-free within one week. The patient was discharged with the advice of follow-up after one month. The clinical course and all investigation findings of this case are presented. Central nervous system tuberculosis may present with non-specific signs and symptoms and may be misdiagnosed as other infections, including dengue, particularly during an ongoing epidemic. It may cause significant morbidity and mortality when the diagnosis is delayed due to its vague clinical presentation.

## Introduction

Tuberculosis is still a leading cause of morbidity and mortality related to infectious disease worldwide, especially in South Asian countries [1]. Though it may involve almost any part of the body, the lungs are the most common site of involvement [2]. In Bangladesh, the prevalence of new pulmonary tuberculosis is about 253 per 100,000 [3]. The central nervous system (CNS) is a less common site of involvement but often has a devastating outcome if not diagnosed and treated early. Patient with CNS tuberculosis usually presents with fever, headache, altered mental status, and weight loss. CNS tuberculosis usually has a subacute or chronic course. Immunocompromised patients are affected more commonly [4]. Patients also usually have other organ involvement like lungs and may have a cough, shortness of breath, or haemoptysis. However, in some cases, they may present with atypical presentations like fever for a short duration, nonspecific symptoms like headache, body ache, photophobia mimicking a viral illness [5]. Especially during an ongoing viral fever outbreak like dengue, these nonspecific features may be misleading.

Since 2010, every year during monsoon (June-September), Bangladesh, especially Dhaka city, has witnessed a dengue epidemic [6]. But in 2019, the number of cases raised to an unprecedented level. In August and September 2019, more than 150,000 dengue cases were diagnosed, mostly in Dhaka city [7]. All the hospitals were flooded with dengue patients; many general hospitals were turned into dengue dedicated hospitals. Dengue patients may also present in the neurology outpatient department (OPD) with headaches and photophobia [8]. These nonspecific symptoms may often mask a graver underlying condition like pyogenic meningitis or CNS tuberculosis.

## Case presentation

A 27-year-old male, a post-graduate student, presented to us in the neurology outpatient department, Bangabandhu Sheikh Mujib Medical University (BSMMU) hospital, with a high-grade fever and headache for five days on 29^th^ August 2019, when there was a dengue outbreak. Fever was intermittent, not associated with any chills and rigor, night sweat, or diurnal variation. The highest recorded peripheral temperature was 102-degrees Fahrenheit. He also had a severe generalized headache, including retro-orbital pain with photophobia for the same duration. The systemic inquiry revealed no other abnormalities. He was healthy before the onset of fever and had no history of contact with individuals infected with tuberculosis. His general physical examination findings were normal. Other than photophobia, neurological examinations also revealed no abnormalities. There were no papilledema, choroid tubercle, focal neurological deficits, or signs of meningeal irritation. Other systems examination findings were also normal.

**Figure 1:**
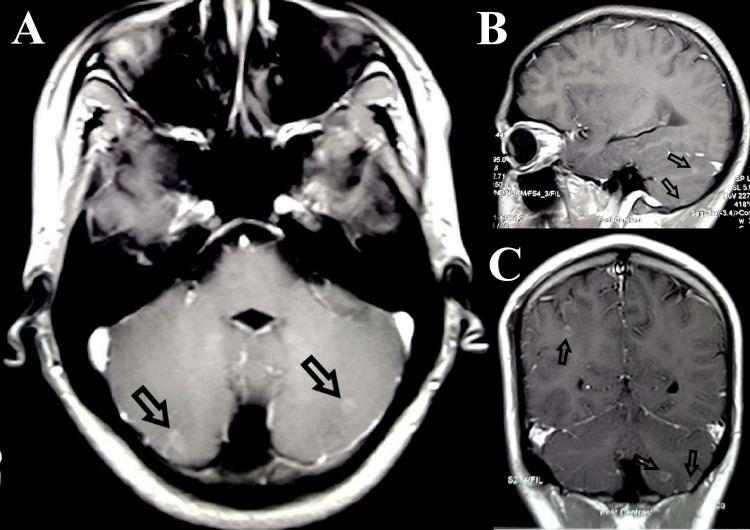
Transaxial (A), sagittal (B), and coronal (C) post Gadolinium T1 weighted MR image of the brain showing multiple supra and infratentorial peripherally enhancing tuberculomas (arrows)

Initial investigations showed neutrophilic leukocytosis (total count 11.00x10^9^/L with 85% neutrophils) and mild hyponatremia (133 mmol/L). Other baseline tests were normal. NS1 antigen for dengue and anti-dengue antibody were done to exclude dengue, which was negative. The patient was admitted for further evaluation. Blood culture, urine routine examination, and culture revealed no abnormalities. As the patient was having persistent fever, headache, and photophobia with increasing severity of hyponatremia (129 mmol/L) on serial serum electrolyte reports, investigations were planned to exclude meningitis. Magnetic resonance imaging (MRI) of the brain with gadolinium showed both supra and infra-tentorial multiple small (2-4 mm) round lesions, smaller ones enhancing homogenously, larger ones with gadolinium-enhanced rim suggesting tuberculomas (Figure 1). But there were no features suggestive of meningeal enhancement. Cerebrospinal fluid (CSF) study revealed crystal clear color, normal pressure, lymphocytic pleocytosis (total cell 10/cubic mm, 90% lymphocytes) with raised protein (79 mg/dl) and low glucose (2.6 mmol/L, CSF and blood glucose ratio 0.37). Mycobacterium tuberculosis DNA was detected in GeneXpert MTB/RIF assay, and the rifampicin-resistant (rpoB) gene was not detected. CSF Gram stain, acid-fast bacillus (AFB) stain revealed no organisms, and the adenosine deaminase (ADA) test was normal (4.4 U/L). Tuberculin test, chest X-ray, and ultrasonography of abdomen were also normal. HIV was excluded as CNS tuberculosis commonly occurs in immunocompromised patients. Evaluation of hyponatremia, including serum and urine osmolality, urinary sodium, etc., was planned, but the patient refused to undergo further evaluation.

The patient was treated with standard anti-tuberculosis therapy (four-drug fixed combinations) according to his weight with pyridoxine and corticosteroid (intravenous dexamethasone 0.4 mg/kg/day tapering over eight weeks). Within one week of starting anti-tuberculosis drugs and steroids, the patient became afebrile with symptomatic improvements like decreased photophobia and headache. Hyponatremia was corrected gradually by oral salt replacement and decreasing fluid intake (137 mmol/L before discharge). The patient was discharged after one month of admission with advice for follow-up after eight weeks. He was hospitalized for one month to complete the dosage of injectable dexamethasone and closely monitor whether he developed any new symptoms. Throughout his hospitalization period, he developed no other symptoms or signs. The patient came to follow up after two months. He had no complaints and had returned to his usual activities. However, we could not do any follow-up MRI of the brain, as the patient did not give consent.

## Discussion

CNS tuberculosis is a relatively rare presentation of tuberculosis (1% of total cases). It may present in different forms like tuberculous meningitis, tuberculomas, tubercular abscess, cerebritis, encephalopathy, spinal tuberculosis, etc. [4]. But the former two are the most common presentations. On many occasions, patients have both tuberculous meningitis and tuberculomas. The patient usually presents with vague complaints like malaise, anorexia, weight loss, and fever for two to four weeks. Sometimes the patient may present with altered mental status, cranial nerve deficits, headache, or vomiting. As most of the initial presentations are nonspecific, often the diagnosis is difficult, leading to delayed onset of treatment. A diagnostic criterion is formulated for a more accurate diagnosis of tuberculous meningitis incorporating clinical, radiological, and laboratory criteria. Fever for more than two weeks is one of the clinical criteria [4,9]. Our patient presented with fever for only fever days with some nonspecific symptoms and normal physical examination. Such unusual presentations may put the physicians in a more challenging position. Many viral fevers may also present with similar presentations like fever, headache, or photophobia. In most cases, these features are self-remitting.

CNS tuberculosis is usually found in association with tuberculosis involving other sites, mostly the lungs [5]. However, isolated CNS tuberculosis may occur, as in this case. Features of meningism like neck rigidity give important clues. Though we often suspect a patient has CNS tuberculosis clinically, it is difficult to confirm the diagnosis by doing investigations. History of previous tuberculosis or evidence of active tuberculosis in other sites may help in the diagnosis. Chest X-ray, sputum examination, imaging of the abdomen may help to find tuberculosis in other sites. Tuberculin test is usually negative in CNS tuberculosis. Brain imaging gives important clues for diagnosis. MRI of the brain is superior to computed tomography (CT) in this regard. MRI of the brain with gadolinium may reveal meningeal enhancement, which is highly suggestive of tuberculous meningitis. It may also reveal multiple tuberculomas. Initially, small tuberculomas enhance homogeneously, indicating granulomas. Later central necrosis occurs, which does not enhance; rather, the surrounding capsule enhances, leading to a classic ring-like appearance. Brain imaging may also reveal other features like hydrocephalus, cerebritis, or infarction [2,4,5].

CSF study is a mandatory test for the evaluation of CNS tuberculosis unless contraindicated. It may reveal turbid color, increased pressure, increased cell count with lymphocyte predominance, increased protein, low sugar, and low CSF serum sugar ratio (<0.6) [4,5]. AFB stain may reveal the organism, but sensitivity is low [4,10]. Raised CSF ADA level may be a simple but important diagnostic tool. In some studies, more than 90% sensitivity and specificity were found, considering 10 u/l as a cut-off value [11]. Gene Xpert MTB/RIF (Cepheid, California, US) assay of CSF is a rapid diagnostic tool that has low sensitivity but high specificity in diagnosing CNS tuberculosis [12].

Other investigations like complete blood count (CBC), serum electrolytes may give important clues. CBC may reveal variable findings, including neutrophilia [13]. Serum electrolytes may reveal hyponatremia, in about 45% of cases of tuberculosis meningitis, due to syndrome of inappropriate secretion of antidiuretic hormone (SIADH), cerebral salt wasting syndrome, adrenal insufficiency, or excess vomiting [14]. Hyponatremia needs further evaluation and specific treatment to prevent complications like osmotic demyelination syndrome.

The prognosis of CNS tuberculosis depends upon the stage of the disease. Early diagnosis and treatment are the most important factors to prevent long-term morbidity and mortality. If the patients present in stage 2 or 3, often the prognosis is poor [4,5]. Fortunately, we started treatment in stage 1 in this case, and the response to treatment was excellent. A combination of antibiotics rifampicin, isoniazid, pyrazinamide and ethambutol for two months followed by the continuation of treatment with rifampicin and isoniazid for 7 to 10 months is the standard treatment globally. At least one year of treatment is necessary for complete recovery. Intravenous dexamethasone 0.4 mg/kg/day, tapering over eight weeks is an important adjunctive treatment in CNS tuberculosis.

## Conclusions

Tuberculosis is an ancient disease but still a challenge despite the availability of modern facilities. A high degree of clinical suspicion is needed in special scenarios like this one for early diagnosis. Even in cases of epidemics of one disease, atypical presentations of another common disease should be considered, and a differential and workup should be done if initial investigations rule out the first disease.
